# Great expectations: minor differences in initial instructions have a major impact on visual search in the absence of feedback

**DOI:** 10.1186/s41235-021-00286-1

**Published:** 2021-03-19

**Authors:** Patrick H. Cox, Dwight J. Kravitz, Stephen R. Mitroff

**Affiliations:** Department of Psychological and Brain Sciences, The George Washington University, Washington, DC USA

**Keywords:** Visual search, Expectation, Instructions, Feedback, Self-fulling prophecy

## Abstract

Professions such as radiology and aviation security screening that rely on visual search—the act of looking for targets among distractors—often cannot provide operators immediate feedback, which can create situations where performance may be largely driven by the searchers’ own expectations. For example, if searchers do not expect relatively hard-to-spot targets to be present in a given search, they may find easy-to-spot targets but systematically quit searching before finding more difficult ones. Without feedback, searchers can create self-fulfilling prophecies where they incorrectly reinforce initial biases (e.g., first assuming and then, perhaps wrongly, concluding hard-to-spot targets are rare). In the current study, two groups of searchers completed an identical visual search task but with just a single difference in their initial task instructions before the experiment started; those in the “high-expectation” condition were told that each trial could have one or two targets present (i.e., correctly implying no target-absent trials) and those in the “low-expectation” condition were told that each trial would have up to two targets (i.e., incorrectly implying there could be target-absent trials). Compared to the high-expectation group, the low-expectation group had a lower hit rate, lower false alarm rate and quit trials more quickly, consistent with a lower quitting threshold (i.e., performing less exhaustive searches) and a potentially higher target-present decision criterion. The expectation effect was present from the start and remained across the experiment—despite exposure to the same true distribution of targets, the groups’ performances remained divergent, primarily driven by the different subjective experiences caused by each groups’ self-fulfilling prophecies. The effects were limited to the single-targets trials, which provides insights into the mechanisms affected by the initial expectations set by the instructions. In sum, initial expectations can have dramatic influences—searchers who do not expect to find a target, are less likely to find a target as they are more likely to quit searching earlier.

## Significance statement

In professions such as radiology and airport security screening, success in visual search—looking for targets among distractors–can be a matter of life-or-death. Therefore, it is critical to understand the factors that influence performance. Many professional searches are carried out in situations where feedback on accuracy is, at best, delayed, but more often never received. When searching in the absence of feedback, the searchers’ initial expectations may play an outsized role in determining search performance. In this study, searchers' initial expectations about the trial distributions, namely the possibility of target-absent trials, were set by a simple manipulation of the wording of the pre-experiment instructions. In the absence of feedback, participants with incorrect low-expectations of a target being present performed less thorough searches than those with correct high-expectations, resulting in more missed targets. This effect was present from the beginning of the experiment such that it created a self-fulfilling prophecy whereby, despite the same true underlying distribution of targets, the low-expectation group actually experienced a lower target prevalence rate. This generated sustained differences in expectations leading to performance differences across the experiment. Interestingly, the effect was specific to the single-target trials; being more likely to find a single target did not make the high-expectation group any better at detecting two targets in the same search display. Beyond serving as a reminder of the importance of carefully crafting instructions for academic experiments, this study highlights the outsized effect that initial expectations can have on search performance in the absence of feedback—a scenario that describes many critical real-world searches. The current work further highlights the importance of setting accurate expectations based on the true target distribution, pointing to the need for professional search industries to fully understand the nature of their environment.

## Introduction

Visual search, finding targets among non-target distractors, is a nearly ubiquitous cognitive act that underlies everyday activities (e.g., finding your keys, looking for a friend in a crowd) as well as highly-important professional tasks (e.g., airport baggage screening, radiology). While efficient and effective performance is useful in everyday searches (no one wants to spend an hour locating their car keys), it is absolutely vital for many professional searches. Taking too long or making mistakes can lead to serious, even life-or-death, outcomes in professional settings such as radiology, pathology, aviation security screening, lifeguarding, border patrol efforts, and various military activities.

Given the prominent role of visual search in so many facets of life, it is not surprising that it has been widely studied (for overviews see Clark et al., 2012; Wolfe, 2020a). Much has been learned about the processes underlying search and the practical factors that affect performance through research in cognitive psychology (for reviews see Chan & Hayward, 2013; Eckstein, 2011 and Nakayama & Martini, 2011), academic radiology (e.g., Krupinski, 2010, 2015; Kundel et al., 1978), aviation security (e.g., Mitroff et al., 2018; Wetter, 2013), military research (e.g., Cornes et al., 2019; Janelle & Hatfield, 2008), and more. Collectively, these efforts have served to isolate core cognitive mechanisms that drive search performance and inform how to optimize performance in a range of practical settings (Wolfe, 2020b).

Since the initial investigations of search (e.g., Koopman, 1956; Poulton, 1890), there has always been a clear and explicit acknowledgement that one of the primary goals of the research efforts is to elucidate factors that impact search performance. Through both theoretical and applied investigations there has been a focus on potential influences, including such factors as the number of non-target items present in the search (e.g., Palmer, 1994), the physical and conceptual relationship between the targets and distractors (e.g., Biggs et al., 2015), external factors such as time pressure (e.g., Pieters & Warlop, 1999) and task goals (e.g., Clark et al., 2014), and individual differences (e.g., Biggs et al., 2013; Boot et al., 2009). Importantly, such factors do not influence performance in isolation—each can impact search on their own but they can also interact with one another in both potentially helpful or detrimental ways.

### Pre-search expectations in the absence of feedback

The current project focused on one particular factor that can influence search performance—searchers’ initial pre-search expectations. Expectations, the belief that something will happen, can be set in a variety of ways for a search task; for example, searchers can be provided with explicit information, including pre-search instructions (e.g., Madrid & Hout, 2019; Yarbus, 1967), cues presented during active search (e.g., Posner et al., 1980), and post-search feedback (e.g., Chabukswar et al., 2003). Likewise, expectations can develop with experience across search experience (e.g., Wolfe et al., 2005); for example, in “contextual cueing” experiments (Chun & Jiang, 1998), searchers become progressively more efficient at finding a target when the same search array is repeated over the course of an experimental session.

Prior research has shown that various forms of expectations can significantly impact search performance. For example, the impact of searchers’ expectations is clear from the fact that when searchers do not regularly see a target, they miss more of the targets that are actually present (e.g., Gur et al., 2004; Wolfe et al., 2005). This “low prevalence effect” has been attributed to both a lower quitting threshold such that participants spend less time searching on trials where there is no target found and to a criterion shift such that the searchers require more evidence to label something as a target (e.g., Wolfe & Van Wert, 2010). That is, when searching in a low-prevalence target environment, the argument is that searchers are quicker to decide that no target is present and they may not conduct an exhaustive search of the whole display and at the perceptual level the decision criterion to call a stimulus a target is stricter (Hout et al., 2015; Godwin et al., 2015). Research into this effect suggests that the prevalence effect gradually builds over recent experience (Ishibashi et al., 2012; Wolfe & Van Wert, 2010).

Interestingly, it has been shown that feedback can impact the effect of low prevalence on expectations. While the majority of the academic work focusing on prevalence effects has provided participants with full and accurate feedback (e.g., Wolfe et al., 2005; Wolfe & Van Wert, 2010; Ishibashi et al., 2012; Ishibashi & Kita, 2014, but see Lau & Huang, 2010 as an example of a study without feedback) one of the exceptions is revealing. Two experiments in Wolfe et al. (2007) presented searchers with a long sequence of low-prevalence search (1% target prevalence) with an occasional burst of high-prevalence search (50% target prevalence) inserted into the sequence. Critically, one of the experiments contained feedback on all trials while the other only included feedback during the high-prevalence bursts. There was negligible impact of the burst of high-prevalence target trials on subsequent periods of low-prevalence search performance in the presence of full feedback, but when accuracy feedback was provided only during the high-prevalence bursts, but not the low-prevalence periods, in the other experiment, there was a significant inoculation against the low-prevalence effect. That is, feedback on accuracy during the high-prevalence bursts created expectations about the underlying target frequency that carried over into the low-prevalence search periods when there was no feedback during those periods to confirm that the underlying target distribution had changed. Relatedly, another line of research has demonstrated that feedback alone can drive changes in search behavior; when participants were presented with false performance feedback they shifted their target-present decision criterion (Schwark et al., 2012), even in the complete absence of actual targets (Schwark et al., 2013).

While much has been revealed about how various forms of expectations can affect search performance, there are open questions that can have meaningful impacts on professional search situations. Specifically, further understanding the impact on search performance of initial expectations (e.g., task instructions) in the absence of feedback is needed given that most professional searches lack timely feedback. Radiologists examining routine cases do not receive real-time feedback as to whether their evaluations are correct or incorrect. Aviation security screeners can potentially receive immediate feedback if they correctly (or incorrectly) detect a prohibited item in a passenger’s bag and pull the bag for secondary search, but they will receive no, or highly delayed, feedback if they do not pull a bag for secondary search.

Arguably, without immediate feedback, professional searchers are largely left to perform “in the dark” where they do not know if their ongoing performance is appropriate or in need of adjustment. Performing a visual search task without feedback can place searchers into a situation where their initial expectations as they start to engage in the search can potentially have an unreasonably large impact on their performance; this is problematic because initial pre-search expectations may not always map correctly onto the reality of the search environment at any given moment. For example, pre-search expectations can change in professional search environments; radiologists can be provided with patient history and aviation security screeners can move between various environments (e.g., the US standard lanes vs. PreCheck lanes). Regardless of context, however, all such searches require a high level of accuracy.

### Self-fulfilling prophecy of search without feedback

Searching without feedback can create a self-fulfilling prophecy where the searchers’ initial expectations going into the search can dramatically shape their actual performance during the search, which in turn serves to bolster their initial expectations. In general, if searchers do not know if targets are present and/or how many possible targets might be present in any given search, then they must adopt a quitting threshold that will determine when they stop looking and move on to the next search (Cain et al., 2012; Chun & Wolfe, 1996). In some situations where accuracy is vital, searchers will ideally adopt a fully exhaustive search strategy where they do not quit the specific search until they have inspected and considered every possible item. However, many factors can lead to non-exhaustive search strategies, including time pressure (Pieters & Warlop, 1999) and expectations (e.g., in low prevalence search; Wolfe & Van Wert, 2010). The choice of a quitting threshold can be driven by feedback; when searchers are provided with trial-by-trial, real time feedback on search accuracy, they can adapt their quitting threshold to appropriately map the structure of their search environment. For example, when receiving feedback in low prevalence search environments that an exceedingly few trials have a target present, searchers will generally speed up and adopt a lower quitting threshold (e.g., Wolfe et al., 2007).

**Fig. 1:**
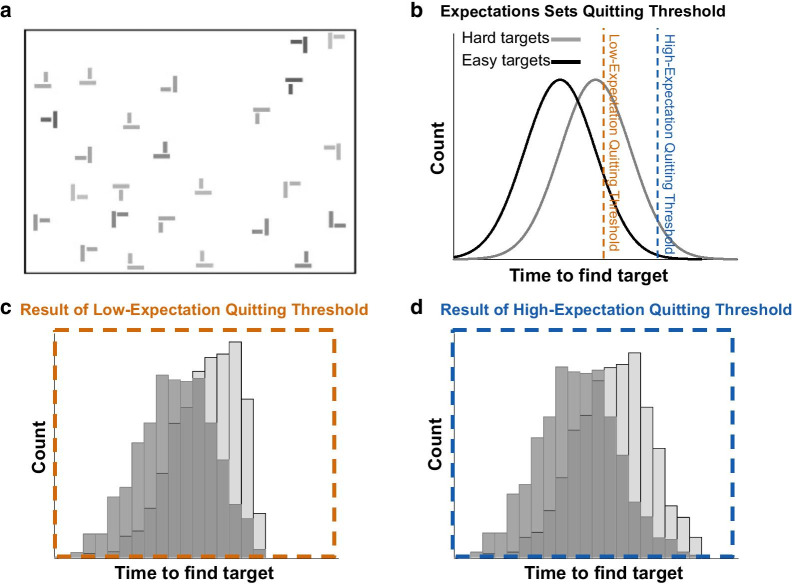
Example stimuli and theoretical model illustrating the predicted results. **a** Example dual-target display. The easy target (dark gray rotated T) is located in the upper left quadrant and the hard target (light gray T) is located in the lower left. **b** Theoretical depiction of underlying difficulty distributions from which the “Hard” (light gray) and “Easy” (dark gray) targets for each trial were drawn from. More difficult-to-find targets fall farther to the right on the distribution and will take longer to find. The prediction is that expectations about the underlying trial structure differentially set the quitting threshold (low-expectation in orange, high-expectation in blue). **c** Hypothetical distribution of found targets for the low-expectation quitting threshold in panel B. **d** Hypothetical distribution of found targets for the high-expectation quitting threshold in panel B. Comparing panels C and D, illustrates the prediction that the higher quitting threshold leads to higher hit rates by allowing time for the searcher to find the more difficult targets from the far right of the distribution in panel B. In turn, the mean response time for finding a target increases with the higher quitting threshold, particularly for the hard targets

Consider a search environment that can contain an equal number of both relatively easy and relatively hard to detect targets (e.g., water bottle vs. bullets in aviation security screenings; high-salience black T vs. low-salience light gray T in Fig. 1a). In such a search environment, the relatively easy-to-spot targets, on average, will be found quicker—but that does not make them any more prevalent; they are just quicker to be found since they stand out more. If the searchers receive real-time accuracy feedback on target detection, then they can learn that easy and hard targets are equally likely in this particular environment, and they may adjust their quitting threshold to search longer when they do not quickly spot an easy target in anticipation that a hard target might be present.

Now, take the above search scenario in the absence of feedback. The searchers, on average, will find the easy targets quicker and more easily, and they will be less likely to detect the hard targets. Without trial-by-trial accuracy feedback, the searchers might incorrectly conclude that easy targets are more prevalent than hard targets, and might adopt a lower quitting threshold such that they will quit the search sooner since their own experiences during the search suggest that targets are likely to be detected quickly.

When search environments do not provide immediate feedback, the searchers’ initial expectations about the nature of the search can create a self-fulfilling prophecy that is divorced from the actual nature of the true search parameters. Continuing with our hypothetical search scenario where there are an equal number of easy- and hard-to-spot targets, imagine there are two groups of participants—the first group a priori expects few, if any, trials to contain a target while the second group expects every trial to contain at least one target. The first group might adopt a lower quitting threshold going into the search and prematurely end their search before fully inspecting every item, while the second group might adopt a higher quitting threshold and will conduct more exhaustive searches (Fig. 1b). The first group will mostly find the easy targets but miss the hard targets (Fig. 1c) while the second group will likely find both easy and hard targets (Fig. 1d). Without feedback, there is nothing to adjust these behaviors and the two groups’ actual experiences will reflect their initial expectations and reinforce their respective initial quitting threshold settings. Low expectation may also lead to a stricter decision criterion for labeling an item as a target (e.g., Wolfe & Van Wert, 2010). This is a distinct possibility given that decision criteria have also been shown to be strongly influenced by feedback (Schwark et al., 2012, 2013).

### Current study

The current project explored how initial pre-search expectations could have a lasting impact on visual search when no feedback is provided. Two groups of searchers completed the same search task, with one sole difference between groups—one sentence of the initial instructions was altered between the groups. One group (“low-expectation” group) was given a sentence in the instructions that incorrectly implied that search trials could have 0, 1, or 2 targets and the other group (the “high-expectation” group) was given a sentence in the instructions that correctly implied that search trials could have 1 or 2 targets. There were no other differences between the two conditions and the critical question was whether this small difference in instructional wording at the start of the experiment could meaningfully impact search performance.

The current hypothesis was that the one-sentence instruction manipulation would set different expectations about the presence of target-absent trials leading the “low-expectation” group to conduct less thorough searches than the “high-expectation” group (Fig. 1b) due to a lower quitting threshold (e.g., Lau & Huang, 2010) and possibly require more evidence to call a stimulus a target than the “high-expectation” group due to a higher decision criterion (e.g., Wolfe et al., 2007). As shown in the theoretical model in Fig. 1, the difference between the two groups was expected to be more extreme on relatively harder trials which should be more affected by variation in quitting thresholds and decision criteria. Further, the current project explored whether this potential effect would manifest as an initial, short-lived difference or whether it would remain as a lasting effect on search performance across the full experimental session. The hypothesis was that despite exposure to the same true distribution of trials, the instruction manipulation would lead to different expectations, and, without feedback on search accuracy, the groups would have different subjective experiences of the same underlying target distribution (Fig. 1). The different subjective experiences would serve as reinforcement of the initial difference in expectation, functioning as a self-fulfilling prophecy whereby the initial difference in expectation is matched in each groups’ experience despite the absence of an actual difference in the true target distribution across groups. This could cause the difference between the groups to remain, or even amplify, across the experiment.

The specific predictions from the hypothesized difference in quitting threshold between the high- and low-expectation groups (Fig. 1b) were fewer timeouts (participants were given a 15 s time limit, see Methods below), lower total search times on miss trials, and, consequently, lower hit rates for the low-expectation group (Fig. 1c, d). The specific predictions from the hypothesized potential difference in the target-present decision criterion were a lower hit rate and lower false alarm rate for the low-expectation group.

## Methods

Two independent participant groups completed the same experimental protocol with the only difference being their initial task instructions. The experimental methods are described in detail below, and they were the same design employed in Adamo et al. (2019). The data for the low-expectation group were previously published as part of a different project with different goals (Porfido et al., 2020; start of semester Spring 2019 cohort).

### Participants

Participants were recruited from The George Washington University’s Psychology department’s subject pool and received course credit. There were two independent participant cohorts recruited at the beginning of the Spring 2019 semester with data collected first for the low-expectation group and then for the high-expectation group: the low-expectation group (*N* = 39, 9 male/ 30 female, mean age 19.67 years, SD 1.47, data collected 1/16/2019–1/28/2019) and the high-expectation group (*N* = 45, 5 male/ 40 female, mean age 19.42 years, SD 1.16, data collected 1/28/2019–2/11/2019). Recent work demonstrated that participants recruited from a course-credit subject pool at the start of the semester performed better at visual search (e.g., were more accurate) and were more compliant than participants from the end of the semester (Porfido et al., 2020). All of the participants in the current study were from the start of the semester (or close to it), and the cohort collected later was the high-expectation group—the cohort predicted to have higher accuracy.

Four additional participants from each group were determined to have contributed outlier data, and their data were removed from all analyses. Outliers were defined as participants whose data were more than two standard deviations from the mean for their own group for easy single-target hit rate (1 low- and 2 high-expectation), false alarm rate (2 low- and 1 high-expectation), or timeout rate (1 low- and 1 high-expectation).

### Stimuli

The stimuli are identical to previously published work (Adamo et al., 2019; different salience condition). Each search display contained 25 items (each 1.3° × 1.3° assuming a 60 cm viewing distance) presented in an invisible 8 × 7 grid (jittered 0–4 pixels from the center of each position within the grid). All items were pairs of perpendicular bars with a small gap between them; targets were perfectly aligned to create a ‘T’ shape and distractors had the crossbars slightly offset by 1–5 pixels from center to create an ‘L’ shape. Each item was presented in one of four rotations (0°, 90°, 180°, and 270°) on a white background. Items were either easy-to-spot high-salience (a grey of 57–65% black; 50% of targets; 5% of distractors) or hard-to-spot low-salience (a grey of 22–45% black; 50% of targets; 95% of distractors). Each display contained one or two targets with the remaining items being distractors. Displays were generated as matched triplets where each dual-target trial (i.e., a trial with two targets present) was made up of an easy target, a hard target, and 23 distractors. Then the matching easy single-target trial was made by converting the hard target into a low-salience distractor and the hard single-target trial was created by converting the easy target into a high-salience distractor. This matched display process was implemented in line with suggestions for properly examining the impact of multiple targets on visual search (Adamo et al., 2019).

### Procedure

Participants were seated approximately 60 cm from a 19-in LCD monitor without head restraint. The stimuli were presented with MATLAB Psychtoolbox (Kleiner et al., 2007). Participants used a computer mouse to click on found targets and pressed the spacebar when finished searching to advance to the next trial. To mimic the time pressures of real world searches, participants had a 15-s time limit per trial—this particular time limit was based on previous studies (Fleck et al., 2010). Failure to complete the search in the time allotted resulted in a timeout warning message. Targets found up to that point were counted as hits and participants then pressed the spacebar to advance to the next trial. Participants started with 12 practice trials, which consisted of a randomized presentation of an equal number of each of the three types of trial (dual-target, easy single-target, and hard single-target) with feedback on hits, misses, and false alarms. Note, the practice trials were made up of the same distribution of trials as the experiment, so neither group experienced target-absent trials in the practice. Participants then completed 9 experimental blocks that each contained 33 trials with no feedback on accuracy (only the timeout warning). Each block contained an equal number of each trial type (dual-target, easy single-target, hard single-target) amounting to 99 total trials of each type across the entire experiment. Displays from the matched triplets were distributed across the first, middle and last third of the blocks in a counterbalanced fashion (See Adamo et al., 2019 for additional details). The dual-target trials played a key role in the procedural manipulation to generate different expectations across groups, but they were secondary for the planned analyses; the primary focus was on first target search performance.

### Critical procedural manipulation of interest

The manipulation of interest in the current study was the pre-experiment instructions provided to the participants. The participants in the *low-expectation group received the following instructions: “On each trial there will be up to 2 targets, meaning you will find 0, 1, or 2 targets.”* The participants in the *high-expectation condition received the following instructions: “On each trial there will be 1 or 2 targets”.* There were no other methodological differences between the two participant cohorts.

### Statistical analyses

Unless otherwise noted, the comparisons of dependent variables of interest across participant groups were carried out using two-sample *t*-tests. The measures of timeout rate, total search time, and false alarm were not normally distributed so the nonparametric Mann–Whitney *U* test was used to compare groups. Chi-squared tests were used to compare the proportion of participants from each group who only missed single targets due to timeouts.

Support for the null hypothesis that there was no difference across the two groups for the accuracy measures on dual-target trials was assessed using a Bayes factor two-sample *t*-test using the *JZS prior* with the scale factor *r* set to 1 (as described by Rouder et al., 2009). Bayes factor analyses were also used to assess non-significant interaction effects in the analyses of instruction group by experimental block. This analysis was conducted using the Bayesian repeated measures ANOVA in JASP (van den Bergh et al., 2019) to compare the posterior probability of a model with the main effects of group and block but no interaction term as the null hypothesis (*H*_0_) to the posterior probability of a full model with the main effects and the interaction term as the alternative hypothesis (*H*_1_). All Bayes factors (*Bf*_01_) are reported as the ratio of the posterior probabilities with the null hypothesis in the numerator, meaning larger numbers correspond to more support for the null hypothesis. Bayes factors greater than 3.2, meaning the null hypothesis is 3.2 times more likely than the alternative, are taken as “substantial evidence” and Bayes factors greater than 10, meaning the null hypothesis is 10 times more likely than the alternative, are taken as “strong evidence” (Kass & Raftery, 1995).

## Results

The primary variables of interest were measures of accuracy (hit rate, false alarm rate) and response time (time to find first target, search termination time on miss trials), as well as timeout rate. The analyses focused on whether performance differed between the participant groups (low-expectation and high-expectation). To anticipate the results, the simple one-sentence difference in the initial pre-experiment instructions led to multiple significant differences that suggest the two groups differed in their quitting threshold and decision criterion for labeling a stimulus a target, but the differences were specific to the single-target trials.

### Timeout rates and search termination times

Evidence for a lower quitting threshold in the low-expectation group compared to the high-expectation group can be seen via analyses of the timeouts and total search time. Participants were to terminate each trial by pressing the spacebar when they had either clicked on each target they found or decided to move on to the next trial. Participants were encouraged not to reach the 15-s timeout limit by issuing a warning that they were too slow if the timeout limit was reached.

**Fig. 2:**
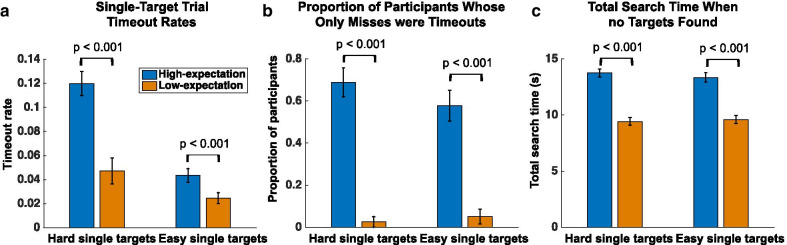
Analysis of timeout rates and search termination times for the high-expectation (blue) and low-expectation (orange) groups. Reported *p*-values are from planned pairwise comparisons. All error bars depict the S.E.M. **a** Timeout rates for hard and easy single-target trials, *p*-values from Mann–Whitney *U* tests. **b** The proportion of participants in each group whose misses were entirely due to timeouts for hard and easy single-target trials, *p*-values from chi-squared tests. **c** Total search times on hard and easy single-target trials where the participants failed to find the target, *p*-values from Mann–Whitney *U* tests

Analysis of the timeout rates by group provided support for an effect of the instructions manipulation on the quitting threshold. The nonparametric Mann–Whitney *U* test was used to compare groups because the timeout rate data were not normally distributed. Consistent with a lower threshold for quitting, the low-expectation group had a significantly lower timeout rate than the high-expectation group for hard single-target trials (low-expectation: mean = 4.72%, SD = 6.76%, high-expectation: mean = 11.99%, SD = 6.74%; *Z* = 4.95; *p* < 0.001) and easy single-target trials (low-expectation: mean = 2.46%, SD = 2.86%, high-expectation: mean = 4.35%, SD = 3.82%; *Z* = 2.73; *p* < 0.01), see Fig. 2a. However, there was no difference in timeout rates on dual-target trials (low-expectation: mean = 1.35%, SD = 2.72%, high-expectation: mean = 1.17%, SD = 1.43%; *Z* = 1.23; *p* = 0.22).

Striking support for an effect of the instruction manipulation on quitting thresholds came from comparing the proportion of participants who only missed targets due to timeouts using chi-squared tests. Specifically, this analysis looked at the rate of participants who never terminated a trial prior to the 15-s limit without finding a target—their only missed targets came from trials in which they searched fruitlessly for 15 s, see Fig. 2b. On the hard single-target trials, 1/39 (2.56%) low-expectation participants only missed targets due to timeouts compared to a much larger proportion of 31/45 (68.89%) for the high-expectation group (*χ*^2^ = 38.97; *p* < 0.001). The same pattern held for the easy single-target trials, 2/39 (5.13%) low-expectation participants only missed targets due to timeouts compared to 26/45 (57.78%) for the high-expectation group (*χ*^2^ = 26.06; *p* < 0.001).

Lastly, total search time (time when the participants terminated the trial by pressing spacebar) was compared between the two participant groups. For this analysis, trials that reached the 15-s timeout limit before the participants terminated the trial were included in the analyses with a value of 15 s since removing the timeout trials would have removed many of the high-expectation participants from the analysis who lacked misses that were not timeouts (see analysis of timeouts above). Note that truncating the total search time distribution by assigning timeouts with a value of exactly 15 s would work against the current hypotheses given that the high-expectation group reached the timeout limit more often. Due to the boundary at 15 s, the total search time data were not normally distributed, therefore the nonparametric Mann–Whitney *U* test was used to compare groups. Total search time for the low-expectation group was significantly shorter on trials without successfully finding a target on both hard single-target trials (low-expectation: mean = 9.43 s, SD = 2.12 s, high-expectation: mean = 13.74 s, SD = 2.40 s; *Z* = 6.34; *p* < 0.001) and easy single-target trials (low-expectation: mean = 9.60 s, SD = 2.15 s, high-expectation: mean = 13.35 s, SD = 2.70 s; *Z* = 5.41; *p* < 0.001; N.B., performance on easy single targets was very high—two participants from the high-expectation and one from the low-expectation group never missed an easy target and could not be included in this analysis), see Fig. 2c. In contrast, when a target was found on single-target trials, there was no significant difference in the total search time between the two groups for either the hard single-target trials (low-expectation: mean = 9.56 s, SD = 1.61 s, high-expectation: mean = 9.03 s, SD = 1.58 s; *Z* =  − 1.27; *p* = 0.20) or the easy single-target trials (low-expectation: mean = 8.88 s, SD = 1.54 s, high-expectation: mean = 8.43 s, SD = 1.70 s; *Z* =  − 1.12; *p* = 0.26). No significant difference in time-to-quit between groups once a target was detected indicates that instruction manipulation successfully set different expectations specifically for 0 vs 1 target, but not 1 versus 2 targets.

### Hit rate and response time

**Fig. 3:**
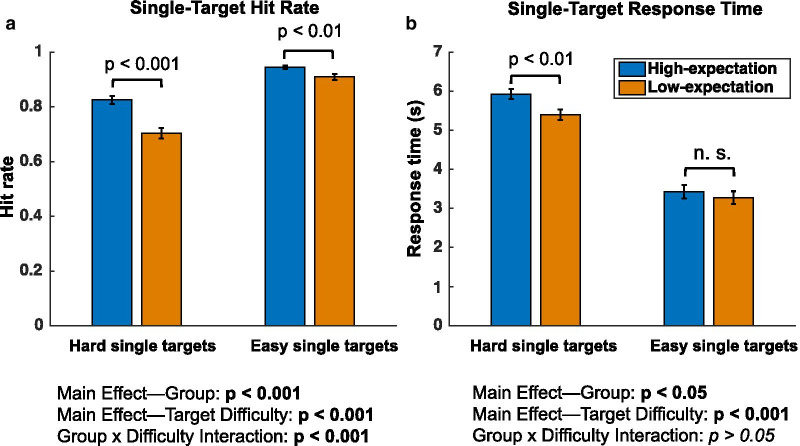
**a** Hit rate and **b** response time data and analyses for easy and hard single-target trials for the high-expectation (blue) and low-expectation (orange) groups. *p*-values are from planned comparisons across groups using two-sample *t*-tests. The error bars depict the S.E.M

Table 1 provides accuracy and response time results and statistical tests comparing the two groups for each measure. For single-target trials, a 2 × 2 ANOVA on hit rate with factors of group (low- and high-expectation) and target difficulty (easy, hard) produced significant main effects of both factors and a significant interaction (main effect of group *F*(1,82) = 23.86, *p* < 0.001, *η*_p_^2^ = 0.225; target difficulty *F*(1,82) = 268.09, *p* < 0.001, *η*_p_^2^ = 0.766; group x difficulty interaction *F*(1,82) = 19.37, *p* < 0.001, *η*_p_^2^ = 0.191). As predicted the low-expectation group had a lower hit rate than the high-expectation group for both hard single-target trials (low-expectation: mean = 70.33%, SD = 11.67%, high-expectation: mean = 82.56%, SD = 9.87%; *t*(82) = 5.20; *p* < 0.001) and easy single-target trials (low-expectation: mean = 90.97%, SD = 7.02%, high-expectation: mean = 94.46%, SD = 4.56%; *t*(82) = 2.73; *p* < 0.01). Furthermore, as predicted, the effect of the instruction manipulation was greater for the hard targets, see Fig. 3a.

**Table 1:** Measures of search performance by group with statistical comparisons

Experimental measure	Low-expectation group	High-expectation group	Statistical comparison
*Total search time*			
Hard single-target misses	9.43 s (2.12 s)	13.74 s (2.40 s)	***Z = 6.34; p < 0.001***
Easy single-target misses	9.60 s (2.15 s)	13.35 s (2.70 s)	***Z = 5.41; p < 0.001***
Hard single-target hits	9.56 s (1.61 s)	9.03 s (1.58 s)	*Z* = − 1.27; *p* = 0.20
Easy single-target hits	8.88 s (1.54 s)	8.43 s (1.70 s)	*Z* = − 1.12; *p* = 0.26
*Timeout rate*			
Hard single-target trials	4.72% (6.76%)	11.99% (6.74%)	***Z = 4.95; p < 0.001***
Easy single-target trials	2.46% (2.86%)	4.35% (3.82%)	***Z = 2.73; p < 0.01***
Dual-target trials	1.35% (2.72%)	1.17% (1.43%)	*Z* = 1.23; *p* = 0.22
*Hit rate*			
Hard single-target trials	70.33% (11.67%)	82.56% (9.87%)	***t(82) = 5.20; p < 0.001***
Easy single-target trials	90.97% (7.02%)	94.46% (4.56%)	***t(82) = 2.73; p < 0.01***
Hard (dual-target trials)	64.89% (12.95%)	64.85% (15.38%)	*t*(82) = − 0.10; *p* = 0.92
Easy (dual-target trials)	88.05% (7.16%)	86.80% (7.01%)	*t*(82) = − 0.81; *p* = 0.42
Dual-target: Both Hit	56.16% (14.23%)	53.09% (18.76%)	*t*(82) = − 0.84; *p* = 0.41
*Hit response time*			
Hard single-target trials	5.40 s (0.84 s)	5.93 s (0.88 s)	***t(82) = 2.80; p < 0.01***
Easy single-target trials	3.28 s (1.02 s)	3.43 s (1.15 s)	*t*(82) = 0.64; *p* = 0.52
*False alarm rate*			
Hard single-target trials	3.16% (6.46%)	5.84% (7.04%)	***Z = 2.78; p < 0.01***
Easy single-target trials	2.88% (6.65%)	3.30% (4.34%)	*Z* = 1.79; *p* = 0.07
Dual-target trials	2.31% (4.49%)	2.31% (3.04%)	Z = 0.39; *p* = 0.70

Significant differences between groups are indicated in bold italics

The 2 × 2 ANOVA on hit rate in the dual-target trials showed no significant main effect of group, no significant interaction, and only a main effect of target difficulty (main effect of group *F*(1,82) = 0.15, *p* = 0.70, *η*_p_^2^ = 0.002; target difficulty *F*(1,82) = 261.64, *p* < 0.001, *η*_p_^2^ = 0.761; group × difficulty interaction *F*(1,82) = 0.11, *p* = 0.74, *η*_p_^2^ = 0.001). Bayes factor analyses on the lack of a difference in the hit rate on dual-target trials for the hard targets (*B*_01_ = 5.95) and easy targets (*B*_01_ = 4.41) found substantial evidence for the null hypothesis in both cases. This lack of a difference on dual-target trials suggests that the instruction manipulation sets differing expectations about the first to-be-found target, specifically, consistent with the instructions differing only in the possibility of 0 target trials occuring.

For response time (time to click on the target) on single-target trials, the 2 × 2 ANOVA produced significant main effects of group and target difficulty, but not a significant interaction (main effect of group *F*(1,82) = 4.31, *p* = 0.041, *η*_p_^2^ = 0.050; target difficulty *F*(1,82) = 276.541, *p* < 0.001, *η*_p_^2^ = 0.771; group × difficulty interaction *F*(1,82) = 1.82, *p* = 0.18, *η*_p_^2^ = 0.191) and a Bayes factor analysis comparing models with (H_1_) and without (H_0_) the interaction term did not support either model (*Bf*_*01*_) = 1.96. Planned post hoc comparisons-revealed that the low-expectation group had significantly faster response times than high-expectation group for the hard single targets (low-expectation: mean = 5.40 s, SD = 0.84 s, high-expectation: mean = 5.93 s, SD = 0.88 s; *t*(82) = 2.80; *p* < 0.01), but did not significantly differ in response time on easy single targets (low-expectation: mean = 3.28 s, SD = 1.02 s, high-expectation: mean = 3.43 s, SD = 1.15 s; *t*(82) = 0.64; *p* = 0.52), see Fig. 3b. Note that the significant difference in response times between the groups for the hard, but not the easy, targets should not be interpreted as a significant difference in the size of the effect of the instruction manipulation for hard vs. easy targets as the interaction term in the group by difficulty ANOVA (*p* = 0.18) was not significant and the Bayes factor for including the interaction was inconclusive (*Bf*_01_) = 1.96). Dual-target response time data in the current study were not particularly informative since the response time for the second target was confounded by the motor response to the first target. As such, they are not discussed further.

### Dual target accuracy and the subsequent search miss (SSM) effect

Previous research has demonstrated that searchers, both professionals and non-professionals, are significantly worse at finding a target if they have already found another target in the same search array (e.g., Berbaum et al., 1990; Biggs & Mitroff, 2015; Fleck et al. 2010). The decreased ability to find a second target after finding a first was originally described in radiology and referred to as “satisfaction of search” (Smith, 1967), but has been more recently been referred to as the “subsequent search miss” (SSM) effect to reflect the multiple underlying cognitive mechanisms at play (Adamo et al., 2013). In the current study, there was no significant difference in dual-target accuracy (defined as the percentage of dual-target trials where both targets where found) between groups (low-expectation: mean = 56.16%, SD = 14.23%, high-expectation: mean = 53.09%, SD = 18.76%; *t*(82) =  − 0.84; *p* = 0.41) and a Bayes factor analysis found substantial evidence in favor of the null hypothesis (*B*_01_ = 4.31). However, measuring the SSM effect involves specifically comparing accuracy for a second-target to a single-target accuracy baseline (a number of different metrics have recently been proposed for measuring the SSM effect, see Adamo et al., 2019; Becker et al., 2020). Therefore, despite the lack of a significant difference in dual-target accuracy, there was a significant difference in the measures of the SSM effect between groups using all of the metrics from both Adamo et al., 2019 and Becker et al. 2020. Critically, these differences were driven by the difference in the *single-target* baseline, so reffering to them as true differences in the SSM effect is complicated (see discussion). For example, a metric comparing the dual-target accuracy to the expected dual-target accuracy derived from the single-target accuracies (the independence assumed metric proposed in Adamo et al., 2019) showed a significant decrement for dual-target performance for both groups (i.e., both groups showed an SSM effect; low-expectation: mean = 8.22%, SD = 8.64%, *t*(38) = 5.94; *p* > 0.001; high-expectation: mean = 25.16%, SD = 13.59%, *t*(44) = 12.42; *p* > 0.001), but that decrement was larger for the high-expectation group (*t*(82) = 6.70; *p* > 0.001).

### Stability of initial expectation effect

**Fig. 4:**
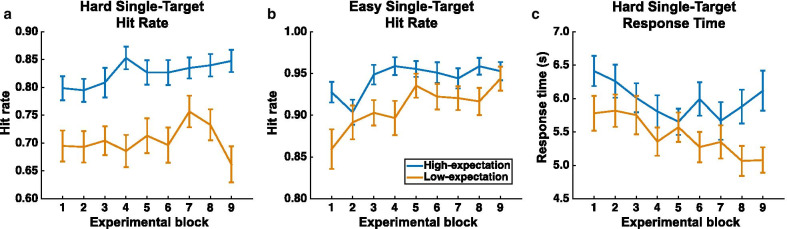
Analysis of the significant effects of high-expectation (blue) versus low-expectation (orange) on hit rate and response time as a function of experimental block to examine the evolution of the effect over time. The difference between groups was present at the beginning of the experiment and remained present over the course of the experiment for **a** easy single-target hit rate, **b** hard single-target hit rate, and **c** hard single-target response time. The group x block interaction effect was not significant for all three measures. Error bars depict the S.E.M

Repeated measures ANOVAs were conducted to examine the evolution of the significant instruction manipulation effects reported above. The instruction manipulation groups served as a 2-level between-subject factor (low-expectation, high-expectation) and the experimental blocks served as a 9-level within-subject factor (blocks 1–9). The primary interest in these analyses were the interaction terms, as significant interactions would suggest that the two groups were differentially affected by experience across the full experimental session while non-significant interactions would be consistent with an initial difference between the groups being maintained across the full experiment. The ANOVA for the hard single-target hit rate showed a main effect of group (*F*(1,82) = 26.58; *p* < 0.001; *η*_p_^2^ = 0.245), but no main effect of block (*F*(8,656) = 1.49; *p* = 0.16; *η*_p_^2^ = 0.018) nor a significant interaction (*F*(8,656) = 1.46; *p* = 0.17; *η*_p_^2^ = 0.018), see Fig. 4a. The ANOVA for the easy single-target hit rate revealed main effects of group (*F*(1,82) = 7.36; *p* < 0.01; *η*_p_^2^ = 0.082) and block (Greenhouse–Geisser corrected; *F*(6.19,507.46) = 5.15; *p* < 0.001; *η*_p_^2^ = 0.059) but no significant interaction (Greenhouse–Geisser corrected; *F*(6.19,507.46) = 1.53; *p* = 0.17; *η*_p_^2^ = 0.018), see Fig. 4b. The ANOVA for the hard single-target response time showed a main effect of group (*F*(1,82) = 10.52; *p* < 0.005; *η*_p_^2^ = 0.114) and block (*F*(8,656) = 2.05; *p* < 0.05; *η*_p_^2^ = 0.024), but no significant interaction (*F*(8,656) = 0.86; *p* = 0.55; *η*_p_^2^ = 0.010), Fig. 4c. Follow up Bayes factor analyses were conducted for each dependent variable of interest comparing a model with only the main effects without the interaction term (H_0_) to a full model including main effects and interactions (*H*_1_). The Bayes factors for each comparison provided strong evidence for the model without the interaction term (hard single-target hit rate: *B*_01_ = 16.68; easy single-target hit rate: *B*_01_ = 12.58; hard single-target response time: *B*_01_ = 89.44). Given there was no effect of the instruction manipulation on the easy single-target response time data, the effect was not examined by block.

The block x group analyses suggest that the instruction manipulation effects on single-target hit rate and response time were present from the beginning of the experiment and did not significantly decrease. That is, despite the fact that participant groups were run on the same underlying distribution of targets, their pre-experiment expectations appear to have set the groups onto different trajectories that were not impacted by the true trial structure. Specifically, this is consistent with the hypothesis that the initial instructions set the participants’ expectations about target prevalence, with the low-expectation group setting a lower quitting threshold and higher target-present decision criterion. Without feedback, this expectation was able to shape the experienced target distribution creating a self-fulfilling prophecy wherein the low-expectation group experienced fewer targets, and fewer hard targets in particular.

### Item-level false alarm rates

The above analyses established an effect of the simple instruction manipulation on search performance, but questions remain about the cause(s) of the effect. The prediction, illustrated in Fig. 1, was that the instruction manipulation would alter participants’ quitting threshold, but it was possible that the participants’ decision criterion could have also been affected when evaluating each individual stimulus within a display (Wolfe & Van Wert, 2010). The current experiment did not include target-absent trials, which does not allow for assessing false alarms at the trial level and signal detection theory analysis. However, the response mechanism did allow for an item-level analysis of false alarms; on each trial participants used the computer mouse to click on any item they felt was a target, which provided an assessment of false alarms as the rate at which they incorrectly identified distractors as targets.

Item-level false alarms were relatively infrequent (Table 1) and false alarm rates were not normally distributed across participants, necessitating the use of nonparametric statistical comparisons. The Mann–Whitney *U* test was used to compute statistically significant differences in false alarm rate between the low- and high-expectation groups across trials. There were significant differences between the low- and high-expectation groups in the percentage of trials that contained item-level false alarms for the hard single-target trials (low-expectation mean = 3.16%, SD = 6.46% vs. high-expectation mean = 5.84%, SD = 7.04%, *Z* = 2.78, *p* < 0.01) and a trend for easy single-target trials (low-expectation mean = 2.88%, SD = 6.65% vs. high-expectation mean = 3.30%, SD = 4.34, *Z* = 1.79, *p* = 0.073). There was no significant difference for dual-target trials (low-expectation mean = 2.31%, SD = 3.04% vs. high-expectation mean = 3.01%, SD = 4.49%, *Z* = 0.39, *p* = 0.70). These results suggest that in addition to a change in quitting threshold, the instruction manipulation might have also led to a shift in the decision criterion.

## Discussion

In this study, two participant groups completed the same simple visual search task, but with one difference in their procedures—the “low-expectation” group was given initial instructions that suggested that there could be no-target trials and the “high-expectation” group that was given initial instructions that suggested that all trials contained at least one target. This singular difference in the initial experimental instructions placed the participant groups on different trajectories from the start based on their pre-search expectations about the nature of the search environment (i.e., whether it would be typical or atypical to fail to find a target on a given trial).

The initial expectations in the absence of feedback created completely different search experiences between the groups. Specifically, a significant change in quitting threshold, as well as a potential change in the target-present decision criterion, made it so that the low-expectation group experienced a search environment in which targets were not always expected, hard-to-spot targets seemed to be more rare, and it was more sensible to quit a trial without having found any target. Without trial-by-trial accuracy feedback, the difference in each group’s initial search expectations led to performance differences that remained across the experiment—regardless of the fact that the groups were doing the identical task.

Interestingly, the effect of the instruction manipulation was specific to the single targets in the current study. While the high-expectation group was more accurate on single-target trials, this advantage did not translate to improved performance on dual-target trials. The lack of an effect on second-target search can most clearly be seen in the equivalent dual-target accuracies between groups. This change in single-target performance without a change in dual-target performance has implications for calculating and reasoning about SSM error rates. Given that measures of the SSM effect (or multiple target search difficulty in general) compare dual-target performance to a single-target baseline (Adamo et al., 2019; Becker et al., 2020), the SSM metrics showed a larger effect for the high-expectation group than the low-expectation group despite no difference in the dual-target performance. Whether or not this should be truly classified as an increase in the SSM effect is a potentially interesting point for debate for those interested in properly quantifying SSM errors. This difference in the measured SSM effect driven by higher single-target performance for the high-expectation group is clearly a different scenario than if the difference in the SSM effect were due to lower dual-target performance for the low-expectation group. Since the SSM effect is theoretically meant to represent a decrement in second-target performance caused by the finding of a first target, it, arguably, does not seem appropriate to classify the current results as an increase in the SSM effect given the equivalent dual-target performance.

A potential limitation of the current finding is that the instruction manipulation could have operated in a categorical fashion; it is possible that the participants were either expecting no-target trials (high-expectation group) or not (low-expectation group). As such, it is an open question how the findings would extend to a situation where the expectations were manipulated in a more graded fashion. For example, it would be interesting to examine the current instruction manipulation when there were no-target trials actually present. Likewise, it would be possible to set the expectations as a higher vs. lower probability of no-target trials rather than all-or-none. Experiments exploring these conditions were underway, but data collection was interrupted by the Covid-19 pandemic. Preliminary data with no-target trials present and the 0, 1, or 2 instruction and no feedback indicated there might be a continuum of expectation based on the distribution of targets found rather than the true underlying distribution in line with the current results and predictions. Further interesting extensions of these findings could involve manipulating time pressure by changing or removing the time limit, by instantiating a reward structure for speed, or by punishing participants for timeouts.

While the current study demonstrated significant differences in performance based on initial expectations, not every search setting will be equally susceptible. There are a wide variety of professional, and non-professional, settings that involve visual search, and each has its own set of particular circumstances. For example, if a search is easy enough that, despite any initial expectation differences, target accuracy is near perfect, then initial differences in expectations would not lead to a difference in experience of the underlying target distribution, and any initial differences in search times would likely dissipate over time. However, many critical professional searches are quite difficult with imperfect accuracy (e.g., Pinto & Brunese, 2010), suggesting this would not be a mitigating factor in many cases.

Overall, the current results are rather straightforward—in the absence of corrective feedback, searchers will experience a self-fulfilling prophecy wherein their own expectations actually mold their experiences. More broadly though, this project is a stark reminder of the importance of subtle differences of instruction wording. A single sentence difference in the initial instructions led to different expectations, which, in turn, led to significantly different search behavior. This has clear implications for research efforts, emphasizing the need to carefully and intentionally determine the instructions for each experiment as a seemingly subtle change could produce wildly different outcomes. Importantly, such meaningful differences from a single wording change in instructions also highlights the complex nature of replications from one lab to another (Open Science Collaboration, 2015).

Beyond the lab, the current results have implications for professional search scenarios that cannot provide immediate case-by-case accuracy feedback. In such environments, It is important to consider professional searchers’ incoming expectations when they are not provided with real-time feedback, as their expectations can guide their self-chosen quitting threshold and decision criterion. Moreover, care must be taken when requiring a professional searcher to move from one search scenario to another, because it may not be trivial to instantaneously update internal expectations and to set appropriate search parameters. For example, radiologists may be provided with patient history, which can create different expectations that result in changes in detection accuracy (e.g., Carney et al., 2012). In routine mammography exams, only 0.5% of the cases will have cancer present (Breast Cancer Surveillance Consortium, 2009), however if the patient has the BRCA1 or BRCA2 genetic mutation (colloquially referred to as the “breast cancer gene”), there is a 82% chance of developing cancer during their lifetime (King et al., 2003). These are dramatically different expected prevalence situations, which can impact the radiologist’s expectations going into the read.

Another implication of the current work for professional searches stems from the fact that while the two participant groups differed in their expectation based on the initial instructions, one was actually accurate as there was in fact a target on every trial. This highlights the point that with appropriate information about the nature of the environment, searchers’ expectations can potentially have a beneficial effect. That is, if searchers have accurately set their expectations, they can perform well in their particular environment even in the absence of case-by-case feedback. However, to provide the searchers with accurate information, each professional search industry needs to have “ground truth” information about their particular setting. The more that is known about the rate and nature of potential targets, the better that training efforts can be designed to empower the workforce to be efficient and effective. Industries may be well-served to do a self-study to determine the ground truth for their particular environment and then use this knowledge to make the best attempt at setting appropriate expectations.

## Data Availability

The data and materials are available upon reasonable request to the authors.
